# Measurement and Time Response of Electrohydrodynamic Direct-Writing Current

**DOI:** 10.3390/mi10020090

**Published:** 2019-01-26

**Authors:** Gaofeng Zheng, Wendong Xue, Huatan Chen, Lingling Sun, Jiaxin Jiang, Xiang Wang, Shumin Guo, Wenwang Li

**Affiliations:** 1 Department of Instrumental and Electrical Engineering, Xiamen University, Xiamen 361102, China; xwd@xmu.edu.cn (W.X.); 35120181152283@stu.xmu.edu.cn (H.C.); lan0xuan@163.com (L.S.); jiangjx@xmu.edu.cn (J.J.); 2 Shenzhen Research Institute of Xiamen University, Shenzhen 518000, China; 3 School of Mechanical and Automotive Engineering, Xiamen University of Technology, Xiamen 361024, China; wx@xmut.edu.cn (X.W.); xmlww@xmut.edu.cn (W.L.); 4 Fujian Provincial Key Laboratory of Mathematical Modeling and High Performance Scientific Computing, School of Mathematical Sciences, Xiamen University, Xiamen 361005, China; shumin_guo@xmu.edu.cn

**Keywords:** electrohydrodynamic direct-writing, direct-written current, micro/nano current measurement, electrical characteristics, jet stability

## Abstract

The micro/nano current is an important characteristic to reflect the electrohydrodynamic direct-writing (EDW) process. In this paper, a direct-written current measurement system with a high signal to noise ratio was proposed to monitor the charged jets, providing the data basis for the promotion of stability and precision of the EDW jet. The electrical characteristics of the printing process were studied, the electrohydrodynamic direct-written current was associated with the stability of charged jet and the accuracy of direct-written patterns. There was an impulse current when the front end of the jet reached the collector and then a stable jet could be gained. With the increase of applied voltage, the severe fluctuation of measured current increased, the charged jet became more unstable and the accuracy of direct-written parallel lines was lower. The effects of processing parameters on direct-written current were also investigated. The average direct-written current at the stable stage increased as the applied voltage and polymer concentration increased, and it decreased as the distance from the nozzle to the collector increased. This research will promote the development and applications of EDW technology in the fields of micro/nano manufacturing.

## 1. Introduction 

With the advantages of easy integration and high deposition accuracy, electrohydrodynamic direct-writing (EDW) [1,2] has displayed great potential applications in various fields, such as flexible electronics [3,4], nanofluidic chips [5,6], and micro/nano sensors [7,8]. However, due to the short distance between the spinneret tip and the collector, the large electrical repulsive force is a big barrier to maintain a stable charged jet, and hinder the promotion of direct-written accuracy [9,10]. Several methods have been proposed to improve the deposition precision of charged jet, including the mechano-electrospinning (MES) [11], pyro-electrohydrodynamic direct-writing [12], and airflow assisted electrohydrodynamic direct-writing [13,14], et al. How to monitor the jet behaviors and promote jet stability has been the key to the industrial application of EDW. 

During the process of EDW, the polymer solution is stretched into a fine jet, and the charges are transferred along the jet to form an electrical current [15,16], which reflect the electrical characteristics and ejection behaviors of charged jet directly. The current mechanisms during the electrohydrodynamic printing process have been studied [17,18,19]. Fridrikh et al. [20] presented a simple model to describe the forces that determine the jet diameter during electrospinning, by which the effect of processing parameters on electrical current was studied. The work indicated that the direct-written current was a special way to reflect the variation of nanofibrous diameter and morphology, which was also good feedback for the precise controlling of the EDW system. Bhattacharjee et al. [21] indicated that the current measured during stable EDW process comprised two components of Ohmic bulk conduction current and surface convection current. The Ohmic bulk conduction current varied with the electrical field strength linearly. However, the surface convection current was independent with the electrical field strength, and varied with the conductivity of fluid and the flow rate linearly. Wang et al. [22] reported that the various electrohydrodynamic ejection modes could be distinguished and recognized based on the current signal, including the modes of dripping, pulsed jet, stable jet, etc. in electrohydrodynamic printing. Munir et al. [23] developed a constant current electrospinning system by using a proportional-integral-derivative (PID) controller, by which the high quality and uniform nanofibers could be produced successfully. 

Attributed to the short nozzle-to-collector distance and low applied voltage, the direct-written current is only tens of nanoampere to several microampere, which is easily to be covered by the system noise. Besides, there would be a complex surrounding electromagnetic field and the ejection and deposition behaviors of charged jet are disturbed easily by the surrounding interferences, introducing more noise into the measurement of direct-written current, which makes it difficult to realize the accurate detection of current signal. As a direct visible characteristic of charged jet, the accurate detection and measurement of at a low noise level could reflect the jet behavior more exactly, which can be used for the precise position controlling of direct-written micro/nano structure. Accordingly, it is urgent to realize the real-time accurate measurement of micro/nano direct-written current for the industrial application of EDW technology.

In this paper, a micro/nano direct-written current measurement system with high signal to noise ratio was developed to investigate the real-time electrical characteristics of direct-written jet and the effect of processing parameters on the direct-written current.

## 2. Materials and Methods 

**Figure 1:**
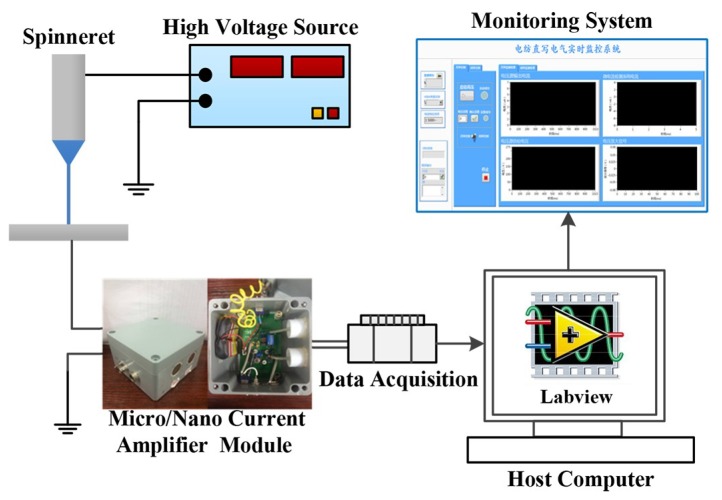
Micro/nano current measurement system of electrohydrodynamic direct-writing.

The micro/nano current measurement system of EDW is shown in Figure 1. A precision syringe pump (Pump 11 Pico Plus Elite, Harvard Apparatus America, Cambridge, MA, USA) was used to supply the polymer solution, by which the solution rate can be adjusted accurately. The anode of the high-voltage DC power supply (DW-SA403-1ACE5, Dongwen high voltage power source Ltd., Tianjin, China) was connected to the stainless-steel nozzle as spinneret, and the cathode was connected to the grounded collector that was fixed on a XY motion platform (GXY1515GT4, Googoltech, Guangzhou, China). To amplify the micro/nano current, a designed micro/nano current amplifier module was connected to the collector, of which the output signal was acquired by a data acquisition card (16 Inputs, Multifunction I/O, National Instruments Corporation, Austin, TX, USA) and transmitted to the host computer in real time. A CCD camera (SSC-DC80, Sony Corporation, Tokyo, Japan) was used to observe the jet motion behaviors.

In these experiments, the polyethylene oxide (PEO, $M_{w}\;$ = 300,000 g/mol, Changchun Dadi Fine Chemical Co., Ltd., Changchun, China) was used as the EDW printing materials. The PEO powder was dissolved in the mixture solvent of deionized water and absolute ethanol (*v*:*v* = 3:1), and the solution was stirred to ensure the complete dissolution of polymer powder.

The micro/nano current amplifier module was designed by current-voltage conversion amplification method, of which the measurement sensitivity was 1 nA, the current range was 1–3000 nA, and the sampling frequency was 1–2000 Hz. Different amplifier circuits were designed to adapt to the measurement requirement for micro/nano current in different scales, of which the amplifier gain was determined by a T-type network resistor when the current was lower than 250 nA or proper feedback resistors selected by a six-bit code switch when the current was higher than 250 nA, respectively. Subsequently, when the direct-written current was lower, the signal was amplified with a high magnification to meet the sensitivity requirement. When the direct-written current was higher, limited by the full scale range of data acquisition card, the feedback resistor could be selected to amplify the signal. 

**Figure 2:**
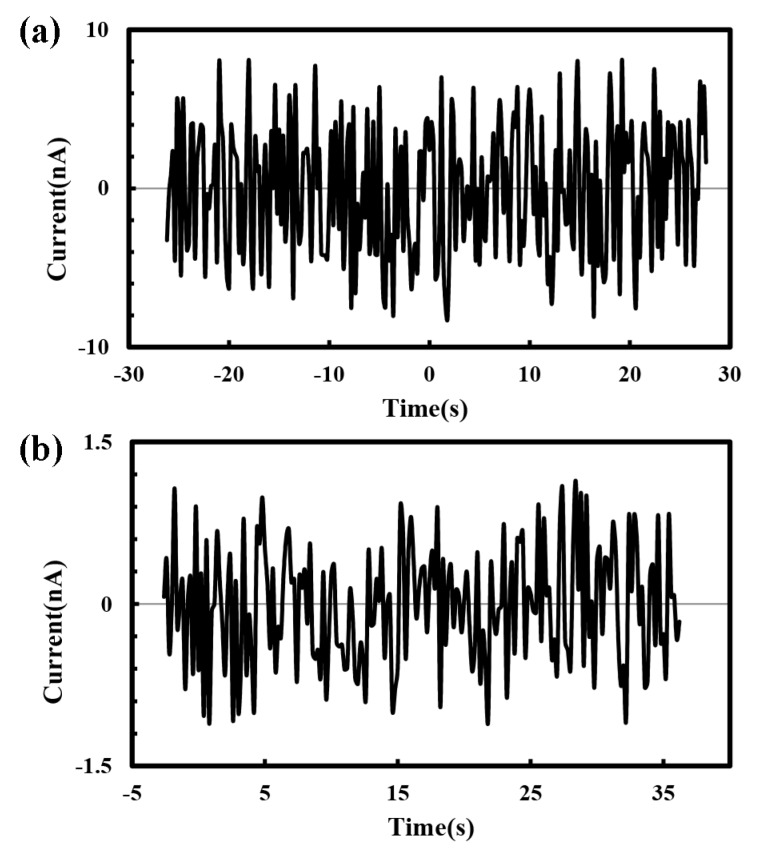
Noise of micro/nano current measurement system without an external load. Current amplitude: (**a**) Lower than 250 nA; (**b**) Higher than 250 nA. The applied voltage and the distance between spinneret and collector were 3 kV and 6 mm, respectively.

Firstly, the signal outputs of the current measurement system without an external load were acquired, which was used to identify the noise amplitude of the micro/nano current measurement system could meet the accuracy requirement. The system noise without an external load for the above two circuits are shown in Figure 2. A high voltage of 3 kV was applied at the time of 0 s. For the current lower than 250 nA, the circuit with T-type network resistor was used and the noise amplitude was about −8 nA–8 nA without a significant fluctuation when the high voltage was applied, as shown in Figure 2a. While for current of 250 nA or higher, a six-bit code switch was used to select feedback resistors with different values to control the current amplification factor. The noise amplitude at different amplification factors were basically maintained at −1.2 nA–1.2 nA without significant differences, as shown in Figure 2b. When compared with the direct-written current signals under the same conditions, the noise amplitude generated by the micro/nano current measurement system was much smaller, which would not disturb the measurement signal of direct-written current. 

## 3. Results and Discussion

**Figure 3:**
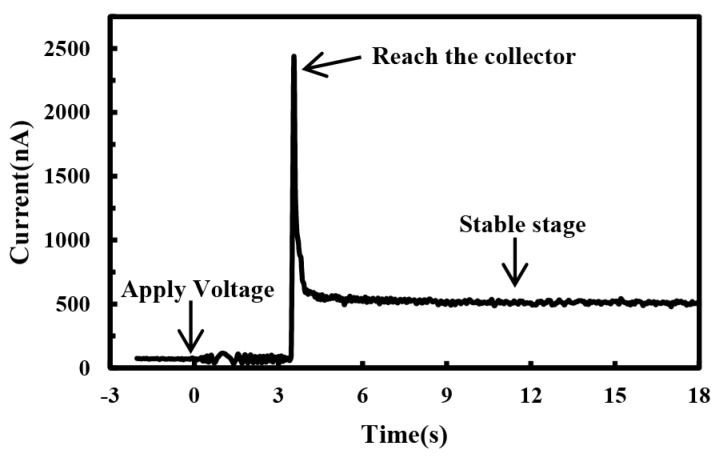
Time response of electrohydrodynamic direct-written current. The applied voltage, the distance between spinneret and collector, the solution concentration and the solution supply rate were 3 kV, 6 mm, 10%, and 100 μL/h, respectively.

The time response of electrohydrodynamic direct-written current is shown in Figure 3. Under the influence of strong electrical field, the current signal fluctuated when the high voltage was applied, and a large amount of free charges accumulated on the polymer droplets at the nozzle. After a certain time delay, sufficient charges were accumulated to overcome the surface tension and viscoelastic force, thus the polymer solution was driven to deform into a jet. When the front end of the jet reached the collector, the free charges that were carried by the liquid jet were conducted to the ground, and a large impulse current signal would occur at this time. The amplitude of impulse current was around 2500 nA. The front end of the jet in the preliminary ejection stage was in bead structure with larger diameter, which was not fully stretched, and the solvent was not fully evaporated. The impulse current occurred when the front end of jet deposited on the collector. Subsequently, the EDW charged jet would step into a stable stage, during which the jet was stretched into finer one and solidified quickly. The charge density on the surface of the stable jet was much smaller than that at the initial stage [24,25], so that the current was decreased significantly. The direct-written current was smaller than the initial peak current. The average current during the stable stage was 500 nA, and the fluctuation range of the direct-written current was 480–540 nA. The current amplitude and fluctuation range were larger than that of system noise without an external load.

**Figure 4:**
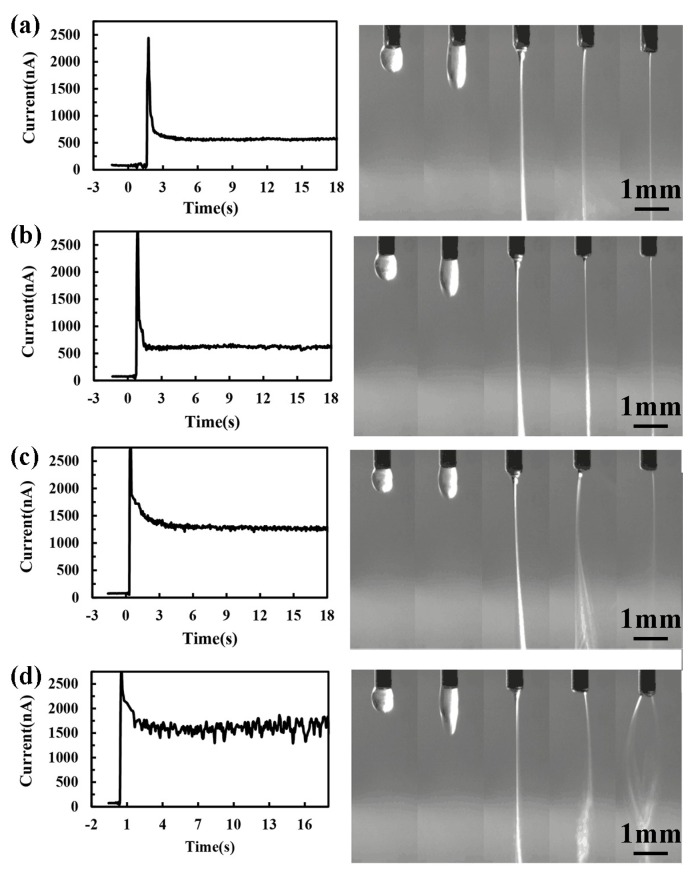
Relationship between electrohydrodynamic direct-written current and ejection time under different applied voltages: (**a**) 3.3 kV; (**b**) 3.6 kV; (**c**) 3.9 kV; and, (**d**) 4.2 kV. The distance between spinneret and collector, the solution concentration and the solution supply rate were 6 mm, 10%, and 100 μL/h, respectively.

**Figure 5:**
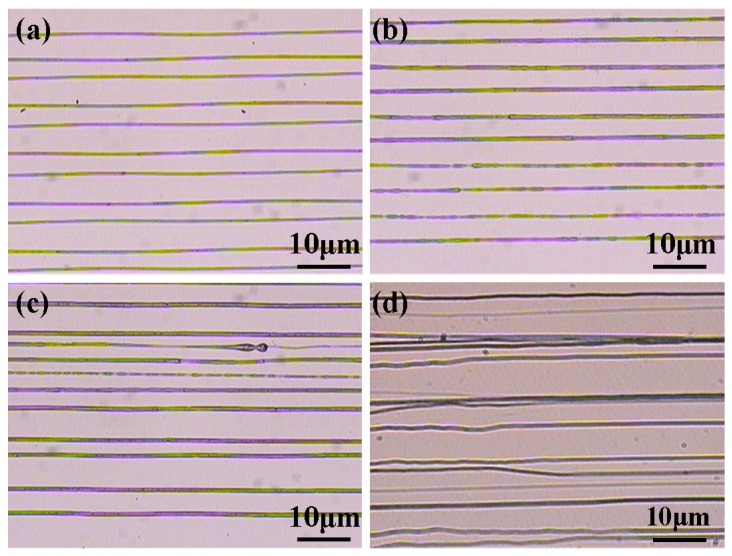
The direct-written patterns under different applied voltages: (**a**) 3.3 kV; (**b**) 3.6 kV; (**c**) 3.9 kV; and, (**d**) 4.2 kV. The distance between spinneret and collector, the collector moving speed, the solution concentration and the solution supply rate were 6 mm, 100 mm/s, 10%, and 100 μL/h, respectively.

Afterwards, the time responses of direct-written current under different voltages were discussed. When the applied voltage was low, the electrical field force played the major role on the jet behaviors and stable jet could be gained. However, with the increase of applied voltage, there would be a large repulsive force between charges, which led to a spiral or bending motion. Therefore, the charged jet became more unstable with the increase of applied voltage, resulting in a more severe fluctuation of measured current, as shown in Figure 4. The electrical field strength and the charge transferring speed increased with the increase of applied voltage, which led to a shorter time for charge accumulation and jet ejection. In this way, the time needed for the jet to step into the stable stage was reduced obviously when the voltage increased from 3 kV to 3.7 kV. The corresponding direct-written patterns were presented in Figure 5. It could be seen that there was a strong link between the direct-written current and the patterns. When the charged jet was stable, the fluctuation of measured current was weak, and parallel lines could be direct-written, as shown in Figure 5a,b. However, when the applied voltage increased to 3.9 kV, with the increase of charge repulsive force, the jet was more unstable, and the fluctuation of measured current was more severe, resulting of a low-resolution of direct-written patterns. When the applied voltage increased to 4.2 kV, the jet whipped strongly, and the fluctuation range of the measured current was severe enough, which corresponded to the disorder deposition of nanofibers. In this way, the measured direct-written current obtained by the developed micro/nano current measurement system was an important value to characterize the EDW process.

**Figure 6:**
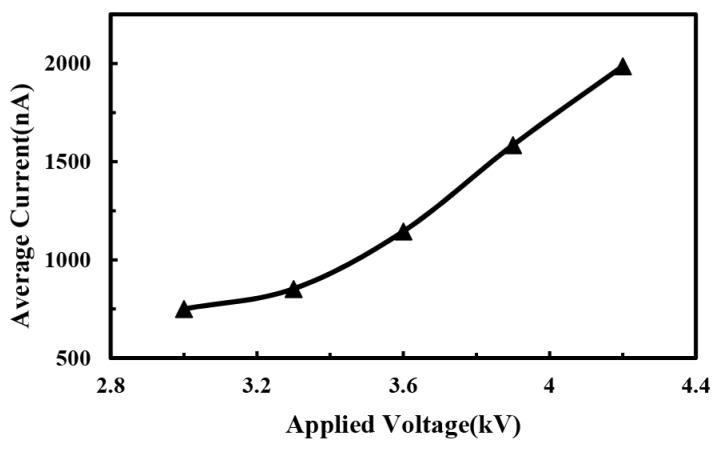
Relationship between average electrohydrodynamic direct-written current and applied voltage. The distance between spinneret and collector, the solution concentration and the solution supply rate were 6 mm, 12%, and 100 μL/h, respectively.

**Figure 7:**
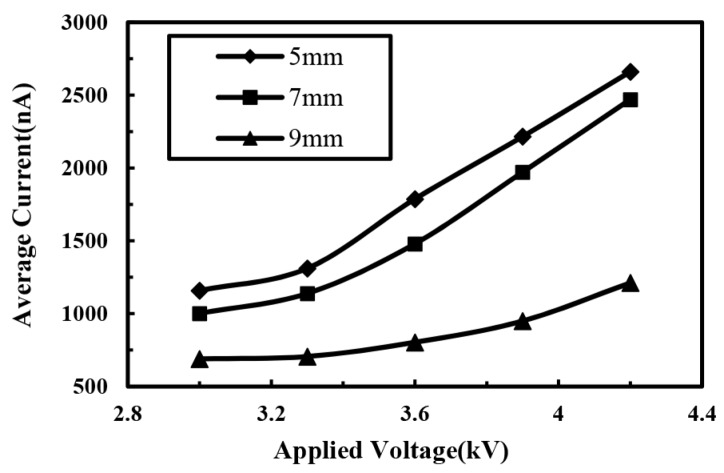
Relationship between average electrohydrodynamic direct-written current and applied voltage at different distances between spinneret and collector. The solution concentration and the solution supply rate were 10% and 100 μL/h, respectively.

In order to further understand the electrical characteristics of the jet, the effects of processing parameters on the average direct-written current were investigated. The relationship between the average direct-written current and the applied voltage is shown in Figure 6. As the applied voltage increased, the intensity of the space electrical field increased, while the surface charge density of the jet and the moving speed of the jet also increased, so that the average direct-written current increased accordingly. When the distance between the spinneret and the collector increased, the spatial electrical field strength decreased, so that the average direct-written current decreased. When the distance was smaller, the effect of voltage acted a more obvious effect on the direct-written current and the direct-written current increased at a higher rate, as depicted in Figure 7. 

**Figure 8:**
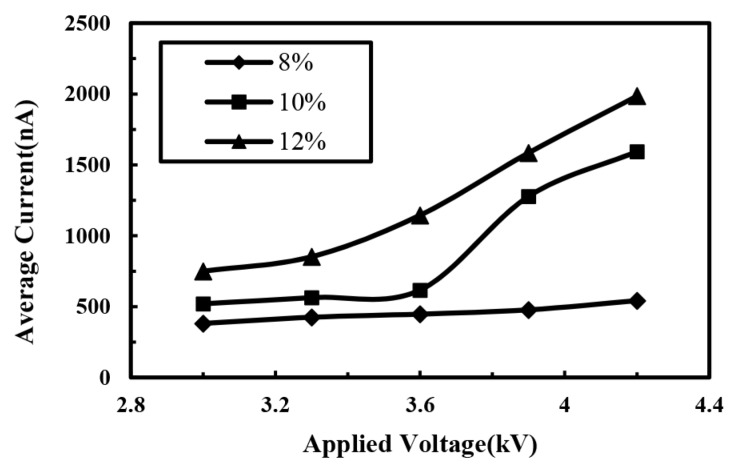
Relationship between average electrohydrodynamic direct-written current and applied voltage under different solution concentrations. The distance between spinneret and collector and the solution supply rate were 6 mm and 100 μL/h, respectively.

The effect of solution concentration was also discussed, as shown in Figure 8. The increase of solution concentration affected the viscosity of the solution, which would increase the viscosity resistance for the jet ejection, decreasing the direct-written current, but also hindering the thinning of the jet. Subsequently, the jet diameter increased, reducing the jet resistance, thus the direct-written current increased reversely. In addition, with the increase of concentration there would be more charges on the jet, which also increased the direct-written current. During these experiments, the average direct-written current increased with the increase of solution concentration, indicating that the decrease of jet resistance and the increase of charge density played a greater impact on the total current. It should be noted that, when the solution concentration was 8%, the effect of voltage on the average direct-written current was not obvious attributing to the fewer conductive ions in the solution. With higher solution concentration, the direct-written current increased at a higher rate.

## 4. Conclusions

In this paper, a micro/nano current measurement system of electrohydrodynamic direct-writing with high noise to signal ratio was developed, providing a quantifiable, high-precision real-time measurement method. The electrical characteristics of electrohydrodynamic direct-writing were characterized. After the high voltage was applied, a large amount of free charges accumulated on the polymer droplets at the nozzle, which was driven to deform into a jet afterwards. Subsequently, the charges were transferred along the jet to form a direct-written current, which was associated with the stability of charged jet and the accuracy of direct-written patterns. In order to further understand the electrical characteristics of charged jet, the effects of processing parameters on the average direct-written current were studied. The results showed that a more intense fluctuation of the current during the stable stage corresponded to a more unstable jet and a lower accuracy of direct-written patterns. As the voltage increased, the electrohydrodynamic direct-written current increased and the time to step into the stable stage reduced. As the distance from the nozzle to the collector decreased, the direct-written current increased, and there would be a more significant effect of the voltage on the current. In addition, the solution concentration also affected the direct-written current under various factors. This work provides the basis to build a close-loop system for the controlling of jet stability and deposition accuracy, which will accelerate the development of EDW technology.

## References

[1] Huang Y., Bu N., Duan Y., Pan Y., Liu H., Yin Z., Xiong Y. (2013). Electrohydrodynamic direct-writing. Nanoscale.

[2] Zheng G., Sun L., Wang X., Wei J., Xu L., Liu Y., Zheng J., Liu J. (2016). Electrohydrodynamic direct-writing microfiber patterns under stretching. Appl. Phys. A Mater..

[3] Wang X., Zheng G., He G., Wei J., Liu H., Lin Y., Zheng J., Sun D. (2013). Electrohydrodynamic direct-writing zno nanofibers for device applications. Mater. Lett..

[4] Zheng Z., Lin G., Zhai T. (2016). Electrospun nanowire arrays for electronics and optoelectronics. Sci. China Mater..

[5] Wang X., Zheng G., Xu L., Cheng W., Xu B., Huang Y., Sun D. (2012). Fabrication of nanochannels via near-field electrospinning. Appl. Phys. A Mater..

[6] Park Y.S., Oh J.M., Cho Y.K. (2018). Non-lithographic nanofluidic channels with precisely controlled circular cross sections. RSC Adv..

[7] Cai X.M., Lei T.P., Sun D.H., Lin L.W. (2017). A critical analysis of the alpha, beta and gamma phases in poly(vinylidene fluoride) using ftir. RSC Adv..

[8] Chang C., Tran V.H., Wang J., Fuh Y.K., Lin L. (2010). Direct-write piezoelectric polymeric nanogenerator with high energy conversion efficiency. Nano Lett..

[9] Zheng G., Li W., Wang X., Wu D., Sun D., Lin L. (2010). Precision deposition of a nanofibre by near-field electrospinning. J. Phys. D Appl. Phys..

[10] Wang Z., Chen X., Zeng J., Liang F., Wu P., Wang H. (2017). Controllable deposition distance of aligned pattern via dual-nozzle near-field electrospinning. AIP Adv..

[11] Bu N., Huang Y., Wang X., Yin Z. (2012). Continuously tunable and oriented nanofiber direct-written by mechano-electrospinning. Mater. Manuf. Process..

[12] Coppola S., Vespini V., Nasti G., Gennari O., Grilli S., Ventre M., Iannone M., Netti P.A., Ferraro P. (2014). Tethered pyro-electrohydrodynamic spinning for patterning well-ordered structures at micro- and nanoscale. Chem. Mater..

[13] Zheng J., Kai Z., Jiang J., He G., Lei X., Liu Y., Liu J., Wu D., Zheng G. (2016). Electrohydrodynamic direct-writing orderly pattern with sheath gas focusing. AIP Adv..

[14] Jiang J.X., Wang X., Li W.W., Liu J., Liu Y.F., Zheng G.F. (2018). Electrohydrodynamic direct-writing micropatterns with assisted airflow. Micromachines.

[15] Saville D.A. (1997). Electrohydrodynamics: The taylor-melcher leaky dielectric model. Annu. Rev. Fluid Mech..

[16] Mora J.F.D.L. (2007). The fluid dynamics of taylor cones. Annu. Rev. Fluid Mech..

[17] Li W.W., Wang X., Zheng G.F., Xu L., Jiang J.X., Luo Z.W., Guo S.M., Sun D.H. (2018). Current characteristics of stable cone-jet in electrohydrodynamic printing process. Appl. Phys. A Mater. Sci. Process..

[18] Higuera F.J. (2011). Electric current of an electrified jet issuing from a long metallic tube. J. Fluid Mech..

[19] Verdoold S., Agostinho L.L.F., Yurteri C.U., Marijnissen J.C.M. (2014). A generic electrospray classification. J. Aerosol Sci..

[20] Fridrikh S.V., Yu J.H., Brenner M.P., Rutledge G.C. (2003). Controlling the fiber diameter during electrospinning. Phys. Rev. Lett..

[21] Bhattacharjee P.K., Schneider T.M., Brenner M.P., Mckinley G.H., Rutledge G.C. (2010). On the measured current in electrospinning. J. Appl. Phys..

[22] Wang X., Zheng G., Luo Z., Li W. (2015). Current characteristics of various ejection modes in electrohydrodynamic printing. AIP Adv..

[23] Munir M.M., Iskandar F., Khairurrijal, Okuyama K. (2008). A constant-current electrospinning system for production of high quality nanofibers. Rev. Sci. Instrum..

[24] Li W., Zheng G., Huang H., Sun D. (2011). Jet current character for electrospinning direct-writing single nanofiber. J. Xiamen Univ..

[25] Zheng G., Sun L., Zheng Y., Li W., Xue W., Zheng J. (2016). Electrical properties of electrospinning under sheath gas focusing. Opt. Precis. Eng..

